# The effect of the implementation of the international code of marketing of breast-milk substitutes on child mortality in Ghana and Tanzania

**DOI:** 10.1017/S1368980024001526

**Published:** 2024-09-24

**Authors:** Juliana Lima Constantino, Stefan Pichler, Lybrich Kramer, Regien Biesma

**Affiliations:** 1 Global Health Unit, Department of Health Sciences, Faculty of Medical Sciences, University of Groningen, Groningen, Netherlands; 2 Department of Economics, Econometrics, and Finance, Faculty of Economics and Business, University of Groningen, Groningen, Netherlands; 3 Department of Nutrition and Dietetics, Hanze University of Applied Sciences, Groningen, Netherlands

**Keywords:** Breast-feeding, Child mortality, Africa, Infant formula, Diarrhoea, Pneumonia, International code of marketing of breast-milk substitutes

## Abstract

**Objective:**

The International Code of Marketing of Breast-Milk Substitutes is an important instrument to protect and promote appropriate infant and young child feeding and the safe use of commercial milk formulas. Ghana and Tanzania implemented the Code into national legislation in 2000 and 1994, respectively. We aimed to estimate the effects of the Code implementation on child mortality (CM) in both countries.

**Setting:**

The countries analysed were Ghana and Tanzania.

**Participants:**

For CM and HIV rates, data from the Institute for Health Metrics and Evaluation from up to 2019 were used. Data for income and skilled birth rates were retrieved from the World Bank, for fertility from the World Population Prospects, for vaccination from the Global Health Observatory and for employment from the International Labour Organization.

**Design:**

We used the synthetic control group method and performed placebo tests to assess statistical inference. The primary outcomes were CM by lower respiratory infections, mainly pneumonia, and diarrhoea and the secondary outcome was overall CM.

**Results:**

One-sided inference tests showed statistically significant treatment effects for child deaths by lower respiratory infections in Ghana (*P* = 0·0476) and Tanzania (*P* = 0·0476) and for diarrhoea in Tanzania (*P* = 0·0476). More restrictive two-sided inference tests showed a statistically significant treatment effect for child deaths by lower respiratory infections in Ghana (*P* = 0·0476). No statistically significant results were found for overall CM.

**Conclusion:**

The results suggest that the implementation of the Code in both countries had a potentially beneficial effect on CM due to infectious diseases; however, further research is needed to corroborate these findings.

Child mortality (CM), which refers to the mortality rates of children below the age of five, has considerably decreased worldwide over the past three decades^(1)^. The number of child deaths has almost halved, from 12·5 million in 1990 to 5 million in 2021^(1)^. Nonetheless, there are large disparities among countries, with more than 90 % of these deaths currently occurring in low- and middle-income countries (LMIC)^(1)^. Around 41 % of these deaths are concentrated in Sub-Saharan Africa, making it the region with the highest CM rates worldwide^(1)^. Despite the shared goal of all nations to achieve United Nations Sustainable Development Goal 3·2, which aims to reduce CM rates to no more than 25 per 1000 births by 2030^(2)^, the average CM rates in 2021 were forty-four in Ghana and forty-seven in Tanzania, illustrating the challenges these countries face^(1)^.

Importantly, approximately 62 % of all child deaths occur in the first year of life and are largely preventable^(3)^. The main causes of mortality in this population remained mostly the same in the last decades including asphyxia and trauma at birth, congenital diseases, lower respiratory infections (LRI) and diarrhoea^(3)^. In 2019 in Ghana, LRI was ranked the fourth cause of the total number of child deaths while diarrhoea was the seventh^(1)^. In the same year in Tanzania, LRI was ranked as the first cause of the total number of child deaths and diarrhoea disease was the tenth^(1)^.

Measures that promote and protect breast-feeding have a major impact on child survival rates and are an important example of a low-cost intervention that can successfully reduce CM caused by LRI and diarrhoea^(4–6)^. In LMIC, children who are not breastfed are six times more likely to die due to infections in the first 2 months and those who are exclusively breastfed are fourteen times less likely to die than those who are not^(7)^. The WHO estimated that reaching optimal levels of breast-feeding can prevent 823 000 deaths of children under the age of 5 annually, mainly by protecting against infectious diseases, and that almost 14 % of the annual deaths of children under the age of two could have been prevented if universal coverage of breast-feeding was reached in seventy-five high-mortality LMIC^(8–11)^.

Aligned with WHO estimations, a study in Ghana showed that 16 % of neonatal deaths would have been prevented if children were breastfed since the first day of life, and 22 % of all children could have been saved if breastfeeding started in the first hour after birth^(12)^. The rates of exclusive breast-feeding until 6 months of age continuously rose from 1988 to 2008 in Ghana, going from 2.2 % to 62.1 %, and have since decreased to 50 % in 2023^(10,11)^. In Tanzania, exclusive breast-feeding until 6 months of age rates continuously increased from 1991 to 2018, going from 25.5 % to 57.8 %^(10)^. Importantly, considering that most child deaths occur in the neonatal stage^(4,5)^, early initiation of breast-feeding is of utmost importance to decrease CM. Both Ghana and Tanzania have rates of initiation of breast-feeding within the first hour of birth of around 50 %, with the rates being 56 % in Ghana^(10,11)^ and 51 % in Tanzania^(10)^. Besides breast-feeding practices, maternal age, number of children, maternal educational level, socio-economic status, maternal employment, births that occurred in health facilities and exposure to HIV at birth are factors associated with CM in Ghana and Tanzania^(4–6)^.

Besides its positive effect on CM, breast-feeding also provides many other benefits for women, children and society^(7–9,11)^. It assists in birth spacing and decreases the risk of the development of non-communicable chronic diseases, cancer and heart disease for women and non-communicable chronic diseases for children^(13)^. Additionally, it is estimated that inadequate breast-feeding levels led to US$570 billion in economic losses annually^(13)^. Therefore, the WHO and The United Nations Children’s Fund recommend exclusive breast-feeding for the first 6 months of life and complementary breast-feeding for at least 2 years^(14,15)^.

However, < 50 % of babies worldwide are exclusively breastfed during the first 5 months^(10)^ and an important obstacle to reaching optimal levels of breast-feeding is the inappropriate marketing of commercial milk formulas (CMF)^(16)^. While some infant and maternal conditions justify CMF use, almost all mothers are physiologically capable of breast-feeding^(17)^. Moreover, the use of CMF increases the risk of CM,^(18,19)^ and it is linked to risks of biological and chemical contamination and malnutrition, especially in LMIC, where due to financial and informational constraints, CMF might be overdiluted or prepared and stored in unsterile conditions^(18,19)^. Furthermore, inappropriate marketing of CMF fortifies false ideas and decreases women’s confidence to breastfeed, and its use exacerbates environmental damage^(13)^. Nonetheless, CMF is strategically marketed and represents a continuously growing market of US$ 70 billion annually, with sales increasing by 21 % in the last 10 years while the rates of exclusive breast-feeding only discretely increased by 12 % from 2000 to 2019^(19–21)^.

In the 1970s, the inappropriate marketing of CMF was recognised internationally as a major issue during the World Health Assembly, leading to the acceptance in 1981 of an international health policy framework to regulate the marketing of CMF called the International Code of Marketing of Breast-milk Substitutes, hereinafter referred to as ‘the Code’^(22)^. The Code is a set of recommendations to regulate the marketing of CMF, feeding bottles and teats, aiming to protect and promote breast-feeding and the safe use of CMF, including monitoring and implementation of the Code^(22)^. It was adopted by the World Health Assembly in 1981 and has been updated through resolutions ever since. In addition to potentially increasing breast-feeding rates, the Code might decrease CM through different channels, by ensuring high quality of CMF and improving information on child feeding^(18,19)^.

Several international treaties reinforce the Code, specifically, the International Covenant on Economic Social and Cultural Rights^(23)^ alongside General Comments 14^(24)^, the Convention on the Rights of the Child^(25)^ in parallel to General Comments 15^(26)^ and the Convention on the Elimination of All Forms of Discrimination against Women^(27)^. In this way, the Code serves as a legal instrument for the treaties’ implementation. From a human rights perspective, the States that are parties to such treaties assume the obligation to respect, protect and fulfill human rights. In this sense, implementing the Code can be considered a measure to protect the right to health and the rights of the child. Thus, the WHO stimulates national governments to implement the Code in their legislative framework, through national legislation or other applicable measures^(14)^.

The WHO assessed countries’ alignment with the Code in 2022 through a scoring algorithm that gives points according to the national legal measures’ alignment to the Code^(14)^. From a total of 100 points, countries that scored 75 or more were considered substantially aligned, between 50 and 75 moderately aligned, and lower than 50 included ‘some provisions’. Until 2022, twenty-five countries, fourteen of which are in Africa, including Ghana, scoring 75 out of 100, and Tanzania, scoring 78 out of 100, were considered substantially aligned; forty-two were moderately aligned (e.g. Mexico); sixty-nine had some provisions (e.g. The Netherlands) and fifty-eight (e.g. United States of America) had no legal measures^(14)^.

In Africa, three countries implemented measures that are substantially aligned with the Code into national legislation – namely Tanzania, Zimbabwe and Ghana^(28–30)^. The respective national law in Ghana is the Breastfeeding Promotion Regulation from 2000^(31)^, and an independent evaluation showed that the law reduced the marketing of CMF and that it is one of the strongest legislative frameworks in the field^(32)^. In Tanzania, the Food Regulations on Marketing of Breastmilk Substitutes and Designated Products was implemented in 1994, with the National Food Control Commission and Tanzanian Food and Nutrition Centre being responsible for improving awareness, monitoring and enforcement of the law^(33)^. Zimbabwe was not included in the analysis since the political and economic situation in the country^(34)^ hampers the efforts to evaluate the effect of policies on child health outcomes.

Considering that both Tanzania and Ghana are at risk of not reaching the United Nations Sustainable Development Goal of reducing CM rates to at least 25 per 1000 births by 2030^(2)^, there is a need to evaluate the national policies that impact CM, including the national laws implementing the Code. Moreover, empirical evidence on the role of legislation in the Code implementation is scarce, and the direct impact of the implementation of the Code on legislation in CM has still not been investigated^(35,36)^. Filling this gap in knowledge will give robust evidence of the legislation’s role. This will facilitate the allocation of resources and contribute to further strengthening Code implementation and monitoring systems. It will also serve as a first step to comprehending the role of legislation on the Code implementation worldwide which in return will serve as a way for children to enjoy their human right of reaching the ‘highest attainable standard of health’^(23,25)^.

## Methods

### Study design

We used a robust, objective and data-driven quasi-experimental design that is used to estimate the effects of policy interventions that take place at an aggregate level, the synthetic control group method (SCGM)^(37,38)^. The SCGM was described as ‘arguably the most important innovation in the policy evaluation literature in the last 15 years’^(39)^. It creates a counterfactual, the synthetic control group, by combining and giving weights to different populations from a donor pool that did not experience the intervention. The synthetic control group mimics the trends that the treated group would have shown in the absence of the intervention.^(37,38)^.

### Data sources

Data for CM and HIV rates came from the Institute for Health Metrics and Evaluation, an independent global research center from the University of Washington.^(21)^ Data for income and skilled birth rates were retrieved from the World Bank, for fertility from the World Population Prospects, for vaccination from the Global Health Observatory of the WHO and for employment from the International Labour Organization. See online supplementary material, Supplementary Table 1 provides the definitions of the variables included in the analysis.

### Outcome variables

The main outcome variables are CM due to diarrhoea and LRI, and the secondary outcome is overall CM. To corroborate the results, we also performed the analysis for two major causes of CM that are unlikely to be directly affected by the intervention, namely CM by congenital diseases and by asphyxia and trauma at birth^(40)^.

### Statistical analysis

A descriptive analysis of the outcome variables and the predictors was conducted, including the mean, standard deviations (sd), maximum and minimum values for each variable. Next, the SCGM was implemented by selecting a donor pool and constructing a counterfactual, reporting pre-treatment characteristics for treatment and counterfactual, showing the main results graphically and performing placebo tests and showing them graphically. Data analysis was conducted on STATA version 17 (StataCorp LLC).

The SCGM was applied with a donor pool that included twenty African and Latin American countries that implemented no legal measures of the Code according to the latest WHO report^(14)^. Countries in the donor pool had a similar gross domestic product per capita to the intervention groups, as the gross domestic product per capita in 2020 was 2·400 USD in Ghana and 1·100 USD in Tanzania, and countries in the donor pool had a gross domestic product in that year lower than 10·000 USD. The Latin American countries included in the donor pool all have a strong African heritage^(41)^.

The predictors included in the analysis were average daily income per capita, skilled birth, fertility, vaccination and employment rates. For the predictor’s vaccination, skilled birth and HIV, the missing data were imputed by calculating the annual growth rates. We also used the outcome variable as predictors during the first year in the pre-treatment period, the middle year between the start of the pre-treatment period and the start of the treatment and the last year before the treatment. It was demonstrated that this approach is preferred but reduces the importance of other predictors^(42)^.

To construct the counterfactual, the method considers the outcome variable and other variables that are associated with the outcome when weighing the donor pool populations during the pre-treatment period^(37,38,42)^. The countries in the donor pool receive weights that vary from zero to one and sum up to one. When the counterfactual matches the trends of the intervention group in the pre-treatment period, the difference between the trends of the treatment group and the counterfactual in the post-treatment period is interpreted as the impact of the intervention^(37)^.

For such an interpretation to hold, we assumed no spillover effects from the treated unit to the donor pool. This assumption is justified since the laws were implemented nationally, and most countries in the donor pool are geographically distant from the treated units. Additionally, we assumed that there were no unobserved shocks that affected the treated units in a different way than affected the control units in the post-treatment period. The SCGM partly accounts for such shocks since the synthetic control is constructed to simulate the outcome dynamics of the treated unit’s observed and unobserved factors. Thus, an important advantage of the SCGM is that it improves causal inference since it considers the effect of confounders changing over time^(37)^.

The root of the mean squared prediction error (RMSPE) in the pre-treatment period was calculated since it serves as an indicator of the fit of the synthetic control group. An RMSPE/Ȳ pre < 10 % indicates a successful replication of the CM dynamics of the treated units in the pretreatment period. The ratio between post and pre-treatment RMSPEs was calculated as it is an indicator of the size of the treatment effect. In particular, an RMPSE ratio bigger than one (= RMSPE post/RMSPE pre > 1) indicates a larger deviation in the post-treatment period, which might be random or due to the treatment effect. To distinguish between the two and assess inference, placebo tests were conducted. The placebo tests apply the SCGM to each country in the donor pool and the RMSPE ratio reported for the treatment group is compared with the ratio found in the countries that did not experience the treatment. Then the RMSPE ratios for the treated unit and placebo groups were ranked. The *P* value is then calculated by the rank of the treatment estimate for the treated group relative to the number of placebo estimates plus one, with a null hypothesis of no treatment effect^(37)^.

In this study, if the treated unity had the highest rank among all twenty countries (one treated and twenty placebos), the *P* value was 1/21 = 0·0476, allowing us to reject the null hypothesis and conclude that there was a statistically significant treatment effect with a significance level of 5 %. As we did not expect that the intervention would lead to an increase in CM, the results of the one-sided inference tests are assessed first. We also report the results of the two-sided inference tests, which might corroborate the results with more conservative estimates. Lastly, considering that the assumptions of the SCGM hold, the level treatment effect (LTE) was used as an indicator of the size of the treatment effect over all post-treatment periods and provided rough estimates of the number of deaths that were prevented on average per year by the intervention when there were significant treatment effects.

## Results

The mean CM in the treated units and the donor pool was 140·89 deaths per 1000 births (sd 84·58) from 1960 to 2019. The mean CM by diarrhoea and by LRI from 1960 to 2019 was 5·49 (sd 2·97) and 12·46 (sd 5·82), respectively. See online supplementary material, Supplementary Table 2 presents the mean, standard deviations (sd), maximum and minimum values for each variable.

### Ghana

**Fig. 1:**
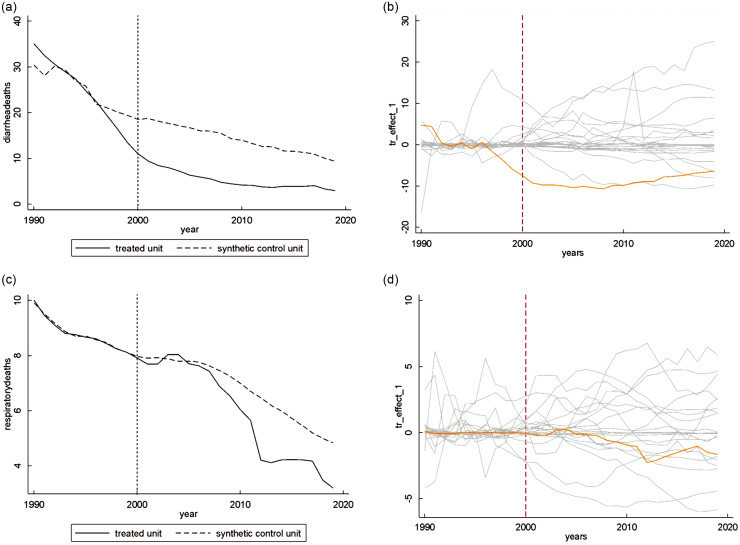
The figure shows the development over time for Ghana and its synthetic control group in terms of child mortality by diarrhea (1A) and lower respiratory infections (1C). The vertical line shows the implementation of the International Code of Marketing of Breast-Milk Substitutes. On the right we depict the difference between Ghana and its synthetic control (orange) and show placebo estimates for the donor pool (gray) to assess statistical significance

The graphical results of the SCGM for breast-feeding-related causes of CM for Ghana are shown in Fig. 1(a)–(d). The solid lines represent the treated unit, while the dashed lines represent the synthetic control group. The composition of each synthetic control group, that is, the unit weights given to each country of the donor pool to create the synthetic control and the predictor rates of the treated and synthetic units, is detailed in see online supplementary material, Supplementary Tables 3 and 4(a) and (b), respectively. The RMSPE in the pre-treatment period varied from 0·02 to 12·21 % of the outcome measure. See online supplementary material, Supplementary Table 5(a) and (b) provides the weights received by the predictors in each analysis.

The graphical results of the placebo tests are shown in Fig. 1(b) and (d). The orange lines represent the difference between the intervention group and the synthetic control group and the grey lines the difference between the intervention group and the placebo units. The synthetic control closely mimics the CM dynamics of the treatment units when the orange line fluctuates closely around the horizontal zero line in the pre-treatment periods.

Figure 1(a) and (b) shows that the rates of CM by diarrhoea started to decrease before the intervention, around 1995. Both one- and two-sided inference tests did not show a statistically significant treatment effect for this outcome, with *P* values of 0·3809 and 0·6667, respectively. The RMSPE pre/Ȳ pre is higher than 10 %, specifically 12·21 %.

Figure 1(c) and (d) illustrates the results for CM by LRI, which start to decrease around 5 years after the intervention. One- and two-sided inference tests showed a statistically significant treatment effect for this outcome, with a *P* value of 0·0476 for both analyses, which had an RMSPE pre/Ȳ pre of 0·56 %.

See online supplementary material, Supplementary Fig. 1(a) and (b) shows the graphical results for CM by congenital diseases, and see online supplementary material, Supplementary Fig. 1(c) and (d) for CM by asphyxia and trauma at birth. One- and two-sided inference tests for both outcomes did not reach statistical significance. The RMSPE pre/Ȳ pre was lower than 10 % for both outcomes, being 0·05 % for congenital diseases and 0·02 % for asphyxia and trauma at birth.

Lastly, see online supplementary material, Supplementary Fig. 2(a) and (b) gives the graphical results for overall CM, which did not reach statistical significance in both one and two-sided inference tests. The RMSPE pre/Ȳ pre for this analysis is lower than 10 %, specifically, 3·75 %. A summary of the results of each analysis is given in Table 1.

**Table 1:** Treatment effects for Ghana

	Ȳ pre	RMSPE pre	RMSPE pre/Ȳ pre (%)	RMSPE post	RMSPE ratio	LTE	Ranking one- sided	*P*-value (one- sided)	Ranking two-sided	*P*-value (two- sided)
	(1)	(2)	(3)	(4)	(5)	(6)	(7)	(8)	(9)	(10)
Child Mortality	164·87	6·19	3·75	3·92	0·63	–2·30	7	0·333	18	0·8571
Diarrhea	25·13	3·07	12·21	8·95	2·91	–8·85	8	0·3809	14	0·6667
Respiratory	8·82	0·05	0·56	1·12	21·11	**–0·84**	**1**	**0·0476**	**1**	**0·0476**
Congenital	4·00	0·21	0·05	0·33	15·46	0·22	20	0·9524	4	0·1904
Asphyxia and Trauma at Birth	11·95	0·25	0·02	1·06	4·12	–0·95	6	0·2857	12	0·5714

**Notes:** The table shows different statistics for Ghana and its synthetic control group. In particular the first column shows the level before the implementation of the International Code of Marketing of Breast-Milk Substitutes. Column (2) shows the root mean square prediction error (RMSPE) between Ghana and its synthetic control group before the implementation. This measure shows a very good fit for all dependent variables in column (3), except for diarrhea. Next the (RMSPE) after the implementation and the RMSPE ratio (after/before) is calculated in columns (4) and (5). Column (6) shows the estimated treatment effect in levels (LTE). This measure is divided by the RMSPE pre (2) and all countries (treated and placebo countries) are ranked based on this ratio. The resulting ranking and *P*-values for one sided tests are shown in columns (7) and (8). Finally, column (9) shows the ranking of the RMSPE ratio (5) for the same set of countries and the resulting *P*-value for the two sided test (10).

### Tanzania

**Fig. 2:**
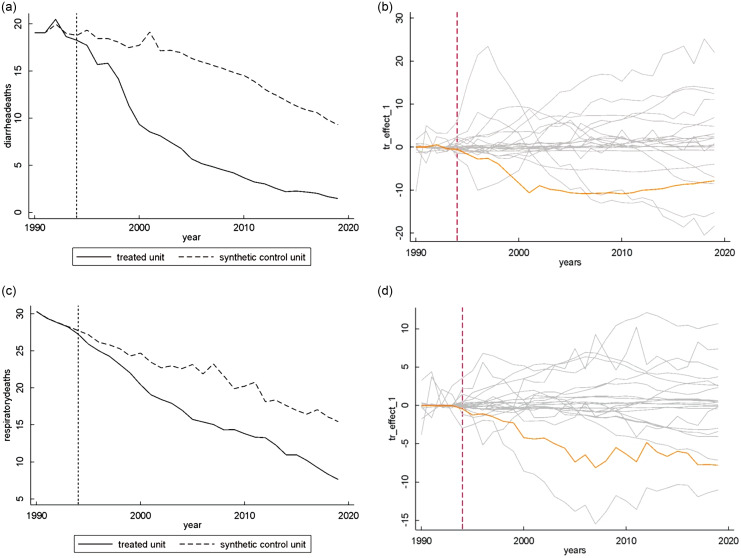
The figure shows the development over time for Tanzania and its synthetic control group in terms of child mortality by diarrhea (2A) and lower respiratory infections (2C). The vertical line shows the implementation of the International Code of Marketing of Breast-Milk Substitutes. On the right we depict the difference between Tanzania and its synthetic control (orange) and show placebo estimates for the donor pool (gray) to assess statistical significance

Figure 2(a) and (b) shows that the rates of CM by diarrhoea started to decrease after the intervention in 1994, and one-sided inference tests showed statistically significant treatment effects for this outcome (*P* = 0·0476; LTE = –8·16). Figure 2(c) and (d) illustrates the results for CM by LRI, which also start to decrease after the intervention, and one-sided inference tests showed statistically significant treatment effects for this outcome (*P* = 0·0476; LTE = –5·16). As many placebo estimates show a large increase in the outcome variables in the post-intervention period, two-sided inference tests did not show significant results for both outcomes.

See online supplementary material, Supplementary Fig. 1(e) and (f) shows the graphical results for CM by congenital diseases, and see online supplementary material, Supplementary Fig. 1(g) and (h) for CM by asphyxia and trauma at birth. One- and two-sided inference tests for both outcomes did not reach statistical significance. The RMSPE pre/Ȳ pre was considerably lower than 10 % for both outcomes, being 0·01 % for congenital diseases and 0·14 % for asphyxia and trauma at birth.

Lastly, see online supplementary material, Supplementary Fig. 2(c) and (d) presents the graphical results for overall CM, which did not reach statistical significance in both one and two-sided inference tests. The RMSPE pre/Ȳ pre for this analysis is lower than 10 %, specifically, 1·32 %. A summary of the results of each analysis is given in Table 2.

**Table 2:** Treatment effects for Tanzania

	Ȳ pre	RMSPE pre	RMSPEpre/Ȳ pre (%)	RMSPE post	RMSPE ratio	LTE	Ranking one- sided	*P*-value (one- sided)	Ranking two-sided	*P*-value (two- sided)
	(1)	(2)	(3)	(4)	(5)	(6)	(7)	(8)	(9)	(10)
Child Mortality	196·97	2·61	1·32	35·86	13·69	–31·70	3	0·1428	4	0·1904
Diarrhea	19·31	0·31	1·61	8·74	27·47	**–8·16**	**1**	**0·0476**	8	0·3809
Respiratory	29·19	0·03	0·10	5·65	187·93	**–5·16**	**1**	**0·0476**	2	0·0952
Congenital	8·07	0·01	0·12	0·53	270·06	–0·41	3	0·1429	3	0·1429
Asphyxia and Trauma at Birth	10·30	0·14	1·36	0·88	62·36	–0·59	15	0·7143	8	0·3809

**Notes:** The table shows different statistics for Tanzania and its synthetic control group. In particular the first column shows the level before the implementation of the International Code of Marketing of Breast-Milk Substitutes. Column (2) shows the root mean square prediction error (RMSPE) between Tanzania and its synthetic control group before the implementation. This measure shows a very good fit for all dependent variables in column (3), as all ratios are smaller than 2 %. Next the (RMSPE) after the implementation and the RMSPE ratio (after/before) is calculated in columns (4) and (5). Column (6) shows the estimated treatment effect in levels (LTE). This measure is divided by the RMSPE pre (2) and all countries (treated and placebo countries) are ranked based on this ratio. The resulting ranking and *P*-values for one sided tests are shown in columns (7) and (8). Finally, column (9) shows the ranking of the RMSPE ratio (5) for the same set of countries and the resulting *P*-value for the two sided test (10).

The LTE of the significant analyses were larger in Tanzania than in Ghana. An LTE of –0·84 and –5·16 was found for CM by LRI in Ghana and Tanzania, respectively, and of –8·17 for CM by diarrhoea in Tanzania. By using the LTE as an indicator of the size of the treatment effect over all post-treatment periods, the number of deaths per 1000 births prevented by the intervention until 2019 was 333 in Tanzania and 16 in Ghana.

## Discussion

This is the first study investigating the effect of the implementation of the Code through national law on CM rates using an innovative analytical tool used to evaluate the effect of policies or legal measures on population health^(37,38)^, the SCGM. One-sided inference tests suggest that the implementation of the Code led to a decrease in CM by LRI in Ghana and Tanzania and by diarrhoea in Tanzania. More conservative two-sided inference tests corroborate the results for CM by LRI in Ghana. As hypothesized, no significant effects were found for CM by birth asphyxia and trauma at birth and by congenital diseases, which corroborate the findings that the Code leads to a decrease in deaths attributed to breastfeeding and child nutrition. This finding also corroborates the non-significant effects for overall CM, which may be attributed to the size of deaths related to nutrition and breast-feeding included in the total number of deaths.

Additionally, the substantial improvements in access to clean water and sanitation in the 90s in Ghana^(43)^ might have had a more pronounced effect on CM due to diarrhoea than the law, which may have contributed to the absence of significant results for this outcome. The short pre-intervention period, in particular for specific causes of child death in Tanzania (from 1990 to 1994), leads to less statistical power since the placebo units might have had a better fit in the pre-intervention period, decreasing the RMSPE ratio. This might have contributed to the absence of an effect on two-sided inference tests in Tanzania.

In both Ghana and Tanzania, birth asphyxia and trauma and congenital birth defects have been among the main causes of CM since 1990^(1)^. Infections do not seem to play a role in deaths related to these conditions, and this lack of influence may have contributed to the absence of significant effects from the Code on overall CM. This finding also highlights the need for interventions in multiple sectors to decrease CM, as child deaths by birth asphyxia and trauma and congenital birth defects mainly involve a combination of adequate prenatal care, skilled medical assistance during labor and delivery, and prompt intervention in case of complications.

Previous research assessing the impact of the laws in Ghana and Tanzania on maternal and child health outcomes and marketing practices related to CMF supports the findings on the potentially positive effect of the Code on CM^(28,44,45)^. The law in Ghana has substantially restricted the promotion of CMF within healthcare facilities and the direct advertisement to the general public and influenced mothers’ feeding options^(28,46)^. Moreover, the establishment of an independent monitoring body in Ghana has enhanced the enforcement of the law^(47)^. Similarly, in Tanzania, maternal exposure to promotions for CMF has been limited since the law implementation^(45)^, and the country has a highly restrictive regulation on CMF promotion on points of sale^(44)^.

It is noticeable that the LTE are larger for Tanzania in comparison with Ghana, which might be a result of the more restrictive enforcement of the law in Tanzania^(44,45)^ in comparison with Ghana^(47)^. This finding might also be a result of different CM rates by LRI in the two countries at the time of the intervention, which were around twenty-seven deaths per 1000 births in Tanzania and eight in Ghana.

By analysing the effects of the Code in two different African countries, non-observed shocks confounding is less likely. Moreover, because Ghana and Tanzania are two diverse countries in the context of Sub-Saharan Africa in terms of cultural and health practices^(4,6)^, the findings can be moderately generalised to other countries in Sub-Saharan Africa with similar socio-economic and legal framework characteristics.

### Limitations

The effects of the Code implementation are difficult to estimate due to challenges in the enforcement and monitoring of the digital marketing of CMF^(13,47)^. In the latest WHO report^(14)^, digital marketing was highlighted as the current dominant platform for marketing in many countries and for which monitoring and enforcement of the Code have been challenging^(20)^. Additionally, the effect of the Code on CM is indirect, by potentially improving breast-feeding rates, the quality of CMF and information on child feeding^(35,36)^.

In cases where mortality rates are not available, the Institute for Health Metrics and Evaluation relies on surveys or indirect estimations based on census or survey data to estimate CM rates. This may impact the comparability of results across countries. However, in LMIC where health statistics are often limited, these data remain the most used source of information on CM rates in the literature^(21,40)^.

For the model with an RMSPE larger than 10 %, namely the analysis for CM by diarrhoea in Ghana, getting significant results is challenging since models with an RMSPE larger than 10 % fails to fully replicate the dynamics of CM in the treated units using synthetic control groups. Additionally, more conservative two-sided inference tests only showed significant effects for child deaths by LRI in Ghana; thus, significant findings for Tanzania need to be interpreted with caution.

A limitation of the SCGM is that it does not allow for completely eliminating any residual confounding or potential impact from other interventions that might have affected the outcome variables, such as the improvements in sanitation previously mentioned for Ghana. Lastly, the results cannot be generalisable to countries outside Sub-Saharan Africa with different socio-economic characteristics and legal frameworks.

### Conclusions

This study underscores the potential beneficial effects of implementing the Code through national laws in reducing CM attributable to infectious diseases. These findings align with previous research^(28,46–48)^. However, it is crucial to acknowledge the limitations to the findings regarding the nature of the SCGM and the available data. Additionally, challenges related to monitoring and enforcing the Code, particularly in the context of digital marketing, must be addressed. Given these considerations, policymakers are encouraged to give serious consideration to the implementation or reinforcement of the Code within their respective countries. Furthermore, complementary measures aimed at safeguarding and promoting breastfeeding should also be explored and implemented. These comprehensive efforts have the potential to bring about substantial improvements in child health outcomes.

## Supporting information

Lima Constantino et al. supplementary material Lima Constantino et al. supplementary material

## Supplementary material

For supplementary material accompanying this paper visit https://doi.org/10.1017/S1368980024001526
