# Supplementary material for: The effect of the implementation of the international code of marketing of breast-milk substitutes on child mortality in Ghana and Tanzania
Source: Public Health Nutr. 2024 Sep 24;27(1):e173. doi: 10.1017/S1368980024001526 (PMC11635799; doi:10.1017/S1368980024001526)
Supplement: Lima Constantino et al. supplementary material [file S1368980024001526sup001.docx]

# Supplementary Tables

**Table 1.** Definition of variables.

| **Variable** | **Definition** |
| --- | --- |
| Child Mortality | 0–5-year-olds dying per 1000 births, data available from 1980 to 2019 |
| Child Mortality by Diarrhea | 0–5-year-olds dying by diarrhea diseases per 1000 births, data available  from 1990 to 2019 |
| Child Mortality by Lower  Respiratory Infections | 0–5-year-olds dying by lower respiratory infections per 1000 births, data  available from 1990 to 2019 |
| Child Mortality by Congenital  Diseases | 0–5-year-olds dying by congenital diseases per 1000 births, data available  from 1990 to 2019 |
| Child Mortality by Asphyxia  and Trauma at Birth | 0–5-year-olds dying by asphyxia and trauma at birth per 1000 births, data  available from 1990 to 2019 |
| Income | Average daily income per capita measured in constant 2011 international $ |
| Fertility | Number of children that were born to each woman |
| Vaccination | Percentage of one-year-olds who received at least one vaccination for  tuberculosis; diphtheria, tetanus toxoid, and pertussis; hepatitis B;  *Haemophilus influenzae* type b; measles; *Porcine circovirus* type 2; polio or  rotavirus |
| Skilled Birth | Percentage of deliveries attended by personnel trained to give the necessary supervision, care, and advice to women during pregnancy, labour, and the postpartum period; to conduct deliveries on their own, and  to care for new-borns |
| Employment | Percentage of females of at least 15 years of age that have been employed during the given year |
| HIV | Estimated percentage of adults aged 15 to 49 years that are infected by HIV, including those without symptoms, those sick from AIDS and those healthy due to treatment of the HIV infection |

**Table 2.** Descriptive analysis of outcome variables and predictors for all countries included in the analysis.

| **Outcome Variables and Predictors** | **From 1960 to 2019** | | | | **From 1990 to 2019** | | | |
| --- | --- | --- | --- | --- | --- | --- | --- | --- |
|  | **Mean** | **SD ^1^** | **Maximum** | **Minimum** | **Mean** | **SD ^1^** | **Maximum** | **Minimum** |
| Child Mortality | 140.89 | 84.58 | 357.00 | 12.70 | - | - | - | - |
| Deaths by Diarrhea | - | - | - | - | 5.49 | 2.97 | 15.74 | 0.17 |
| Deaths by Lower Respiratory Infections | - | - | - | - | 12.46 | 5.82 | 45.93 | 0.56 |
| Deaths by Congenital Diseases | - | - | - | - | 5.20 | 2.41 | 11.80 | 1.77 |
| Deaths by Asphyxia and Trauma at Birth | - | - | - | - | 6.99 | 3.23 | 17.99 | 0.69 |
| Income | 4.90 | 4.44 | 41 | 0.22 | 5.75 | 5.54 | 41 | 0.69 |
| Fertility | 5.32 | 1.54 | 7.69 | 1.42 | 4.54 | 1.55 | 7.69 | 1.42 |
| Vaccination | 77.32 | 20.99 | 99 | 3 | 82.81 | 16.39 | 99 | 21 |
| Skilled Birth | 54.33 | 27.39 | 99 | 4 | 57.62 | 27.09 | 99.9 | 5 |
| Employment | 49.57 | 16.95 | 83.3 | 17.9 | 49.55 | 16.97 | 83.3 | 17.9 |
| HIV | 3.51 | 5.73 | 26 | 0 | 4.22 | 6.04 | 26 | 0 |

1. SD = standard deviations

**Table 3.** Unit weights per analysis

| **Country Code** | **Country Name** | **Ghana and CM ^1^** | **Ghana and**  **Diarrhea** | **Ghana and**  **LRI ^2^** | **Ghana and Congenital** | **Ghana and**  **Asphyxia** | **Tanzania and CM ^1^** | **Tanzania and**  **Diarrhea** | **Tanzania and LRI ^2^** | **Tanzania and**  **Congenital** | **Tanzania and**  **Asphyxia** |
| --- | --- | --- | --- | --- | --- | --- | --- | --- | --- | --- | --- |
| 2 | Central African Republic | 0 | 0 | 0 | 0 | 0 | 0.1 | 0 | 0.243 | 0.6 | 0 |
| 3 | Congo | 0 | 0 | 0 | 0 | 0 | 0.59 | 0 | 0 | 0 | 0 |
| 4 | Eritrea | 0.334 | 0.473 | 0 | 0 | 0.29 | 0 | 0.137 | 0 | 0.015 | 0.157 |
| 5 | Eswatini | 0 | 0 | 0.233 | 0.239 | 0 | 0 | 0.691 | 0.063 | 0 | 0 |
| 6 | Guinea | 0 | 0 | 0 | 0 | 0.265 | 0 | 0 | 0.267 | 0.225 | 0.203 |
| 7 | Haiti | 0 | 0 | 0.189 | 0 | 0 | 0 | 0 | 0 | 0.16 | 0 |
| 8 | Jamaica | 0 | 0 | 0.485 | 0.0701 | 0 | 0.163 | 0 | 0 | 0 | 0 |
| 9 | Lesotho | 0.148 | 0 | 0 | 0 | 0 | 0 | 0 | 0 | 0 | 0.583 |
| 10 | Mauritius | 0 | 0 | 0 | 0.26 | 0 | 0 | 0 | 0 | 0 | 0 |
| 11 | Morocco | 0 | 0.527 | 0 | 0 | 0 | 0 | 0 | 0 | 0 | 0 |
| 12 | Namibia | 0.154 | 0 | 0 | 0 | 0 | 0 | 0 | 0.031 | 0 | 0 |
| 13 | Somalia | 0 | 0 | 0 | 0 | 0 | 0 | 0 | 0 | 0 | 0 |
| 14 | South Sudan | 0.186 | 0 | 0 | 0 | 0.445 | 0 | 0 | 0 | 0 | 0 |
| 15 | Suriname | 0 | 0 | 0 | 0 | 0 | 0 | 0 | 0 | 0 | 0 |
| 16 | Togo | 0 | 0 | 0.093 | 0.036 | 0 | 0 | 0.123 | 0 | 0 | 0 |
| 17 | Angola | 0 | 0 | 0 | 0.394 | 0 | 0 | 0 | 0 | 0 | 0 |
| 18 | Belize | 0 | 0 | 0 | 0 | 0 | 0 | 0 | 0 | 0 | 0 |
| 19 | Equatorial Guinea | 0 | 0 | 0 | 0 | 0 | 0 | 0 | 0.397 | 0 | 0 |
| 20 | Guyana | 0.179 | 0 | 0 | 0 | 0 | 0.027 | 0 | 0 | 0 | 0 |
| 21 | Liberia | 0 | 0 | 0 | 0 | 0 | 0.119 | 0.048 | 0 | 0 | 0.057 |

(1) CM = child mortality; (2) LRI = lower respiratory infections

**Table 4A.** Predictor rates of the treated and synthetic units for Ghana

| **Predictors Treated \|Synthetic**  **Analyses** | **Income** | | **Fertility** | | **Vaccination** | | **Skilled Birth** | | **Employment** | | **HIV** | | **Mortality 1999** | | **Mortality 1980**  **(CM) and 1977**  **(other causes)** | | **Mortality 1960**  **(CM) and 1990**  **(other causes)** | |
| --- | --- | --- | --- | --- | --- | --- | --- | --- | --- | --- | --- | --- | --- | --- | --- | --- | --- | --- |
| Ghana and CM ^1^ | 2.84 | 4.05 | 5.69 | 5.47 | 64.75 | 69.94 | 40.54 | 41.47 | 67.08 | 50.26 | 0.71 | 3.29 | 103 | 103.12 | 166 | 165.09 | 209 | 223.99 |
| Ghana and Diarrhea | 3.25 | 5.99 | 5.04 | 4.29 | 90 | 89.59 | 45.56 | 33.28 | 66.42 | 41.87 | 2.1 | 0.59 | 13.49 | 19.40 | 24.78 | 25.79 | 35.11 | 30.43 |
| Ghana and LRI ^2^ | 3.25 | 5.81 | 5.04 | 3.77 | 90 | 88.00 | 45.56 | 67.82 | 66.42 | 45.33 | 2.1 | 5.59 | 8.12 | 8.12 | 8.67 | 8.71 | 10.01 | 9.91 |
| Ghana and Congenital | 3.25 | 5.01 | 5.04 | 4.69 | 90 | 79.41 | 45.56 | 59.07 | 66.42 | 50.77 | 2.1 | 4.78 | 4.09 | 4.09 | 4.02 | 4.06 | 3.90 | 3.90 |
| Ghana and Asphyxia | 3.25 | 3.06 | 5.04 | 6.17 | 90 | 73.90 | 45.56 | 18.63 | 66.42 | 62.12 | 2.1 | 1.70 | 12.05 | 12.05 | 12.08 | 12.09 | 11.57 | 11.58 |

(1) CM = child mortality; (2) LRI = lower respiratory infections

The first columns give the rates for the treated units, and the second columns for the synthetic control unit

**Table 4B.** Predictor rates of the treated and synthetic units for Tanzania

| **Predictors**  **Treated \| Synthetic**  **Analyses** | **Income** | | **Fertility** | | **Vaccination** | | **Skilled Birth** | | **Employment** | | **HIV** | | **Mortality 1993** | | **Mortality 1995**  **(CM) and 1992**  **(other causes)** | | **Mortality 1960**  **(CM) and 1990**  **(other causes)** | |
| --- | --- | --- | --- | --- | --- | --- | --- | --- | --- | --- | --- | --- | --- | --- | --- | --- | --- | --- |
| Tanzania and CM ^1^ | 1.63 | 3.51 | 6.43 | 6.07 | 86.07 | 59.86 | 39.24 | 61.91 | 81.17 | 64.12 | 4.82 | 3.25 | 161 | 160.94 | 187 | 191.14 | 243 | 243.14 |
| Tanzania and Diarrhea | 1.68 | 1.89 | 6.11 | 5.63 | 93.25 | 84.84 | 43.45 | 49.01 | 81.13 | 41.48 | 6.03 | 3.01 | 18.64 | 19.01 | 20.46 | 19.95 | 19.07 | 19.07 |
| Tanzania and LRI ^2^ | 1.68 | 1.68 | 6.11 | 6.02 | 93.25 | 84.58 | 43.45 | 26.43 | 81.13 | 53.14 | 6.03 | 2.12 | 28.25 | 28.29 | 28.81 | 28.83 | 30.30 | 30.32 |
| Tanzania and Congenital | 1.68 | 1.38 | 6.11 | 5.86 | 93.25 | 78.93 | 43.45 | 37.53 | 81.13 | 60.51 | 6.03 | 3.95 | 7.91 | 7.91 | 8.00 | 8.00 | 8.25 | 8.25 |
| Tanzania and Asphyxia | 1.68 | 3.24 | 6.11 | 5.48 | 93.25 | 76.17 | 43.45 | 46.99 | 81.13 | 48.11 | 6.03 | 2.12 | 10.52 | 10.49 | 10.38 | 10.39 | 10.08 | 10.08 |

(1) CM = child mortality; (2) LRI = lower respiratory infections

The first columns give the rates for the treated units, and the second columns for the synthetic control unit

**Table 5A.** Predictors’ weights per analysis for Ghana

| **Predictors**  **Analyses** | **Income** | **Fertility** | **Vaccination** | **Skilled Birth** | **Employment** | **HIV** | **Mortality 1999** | **Mortality 1980 (CM) and 1977 (others)** | **Mortality 1960 (CM)**  **and 1990 (others)** |
| --- | --- | --- | --- | --- | --- | --- | --- | --- | --- |
| Ghana and CM ^1^ | 0.008 | 0.019 | 0.025 | < 0.001 | 0.016 | < 0.001 | 0.119 | 0.438 | 0.376 |
| Ghana and Diarrhea | 0.002 | < 0.001 | 0.001 | 0.002 | < 0.001 | < 0.001 | 0.174 | 0.592 | 0.228 |
| Ghana and LRI ^2^ | < 0.001 | < 0.001 | 0.003 | < 0.001 | 0.002 | 0.002 | 0.026 | 0.966 | 0.002 |
| Ghana and Congenital | 0.006 | 0.003 | < 0.001 | 0.047 | 0.064 | 0.024 | 0.009 | 0.841 | 0.005 |
| Ghana and Asphyxia | 0.008 | 0.017 | 0.105 | 0.003 | 0.065 | 0.029 | 0.031 | 0.742 | < 0.001 |

(1) CM = child mortality; (2) LRI = lower respiratory infections

**Table 5B.** Predictors’ weights per analysis for Tanzania

| **Predictors**  **Analyses** | **Income** | **Fertility** | **Vaccination** | **Skilled Birth** | **Employment** | **HIV** | **Mortality 1993** | **Mortality 1995 (CM) and 1992 (others)** | **Mortality 1960 (CM)**  **and 1990 (others)** |
| --- | --- | --- | --- | --- | --- | --- | --- | --- | --- |
| Tanzania and CM ^1^ | < 0.001 | 0.006 | 0.042 | 0.029 | 0.377 | < 0.001 | 0.348 | 0.008 | 0.190 |
| Tanzania and Diarrhea | 0.044 | 0.026 | 0.363 | < 0.001 | < 0.001 | 0.003 | 0.328 | 0.118 | 0.119 |
| Tanzania and LRI ^2^ | < 0.001 | 0.014 | 0.039 | < 0.001 | < 0.001 | 0.027 | 0.022 | 0.891 | 0.001 |
| Tanzania and Congenital | 0.088 | < 0.001 | < 0.001 | 0.059 | 0.021 | 0.033 | 0.060 | 0.615 | 0.123 |
| Tanzania and Asphyxia | < 0.001 | 0.001 | < 0.001 | 0.003 | < 0.001 | 0.004 | 0.079 | 0.849 | < 0.001 |

(1) CM = child mortality; (2) LRI = lower respiratory infection

**Figure 1.** SCGM results for non-breastfeeding-related CM causes in Ghana and Tanzania.


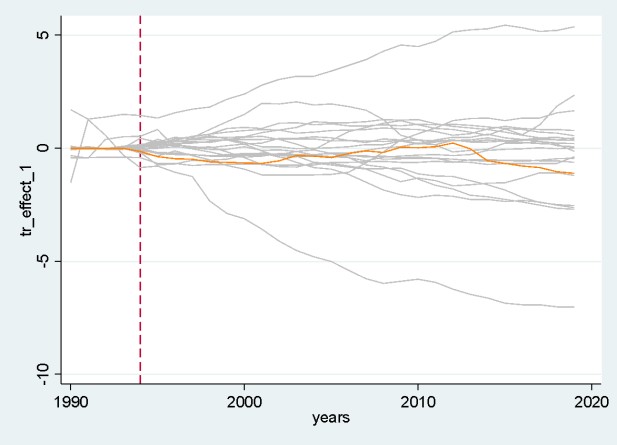

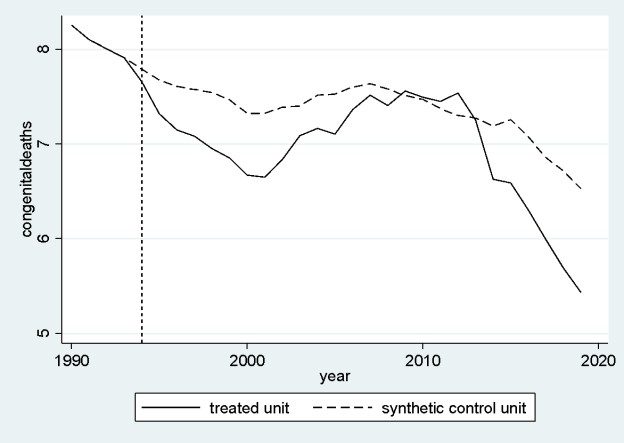

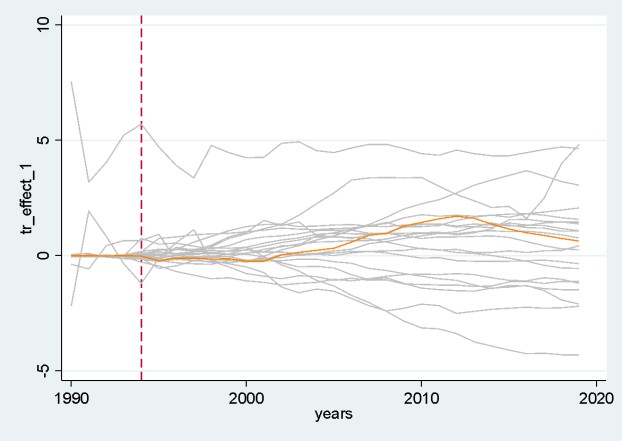

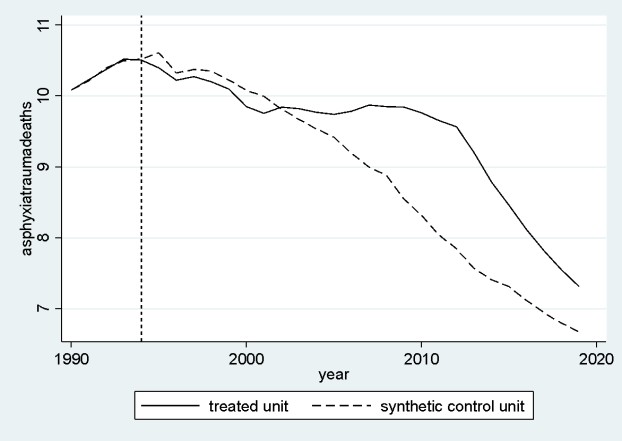


**Figure 1E.** SCGM for CM by congenital diseases in Tanzania

**Figure 1F.** Placebo tests for CM by congenital diseases in Tanzania

**Figure 1G.** SCGM for CM by asphyxia/trauma in Tanzania

**Figure 1H.** Placebo tests for CM by asphyxia/trauma in Tanzania


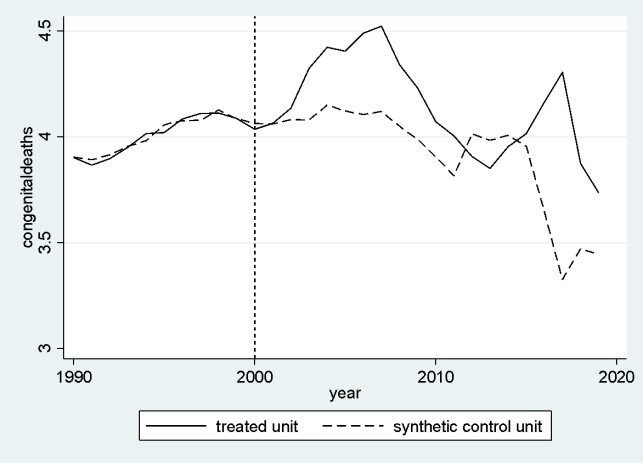

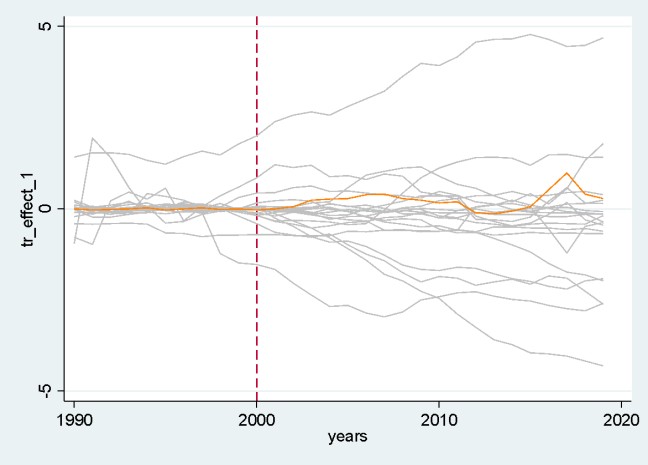

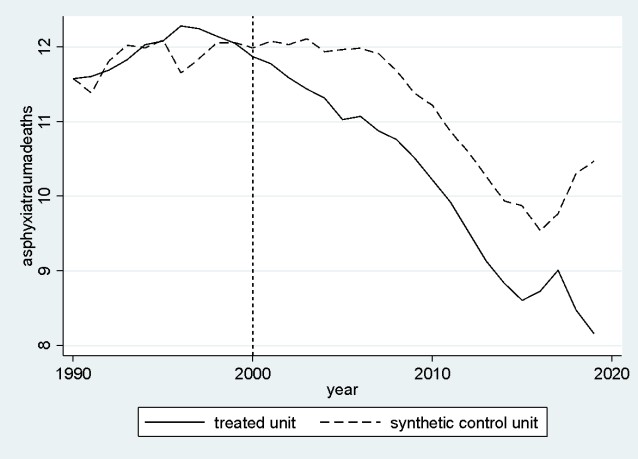

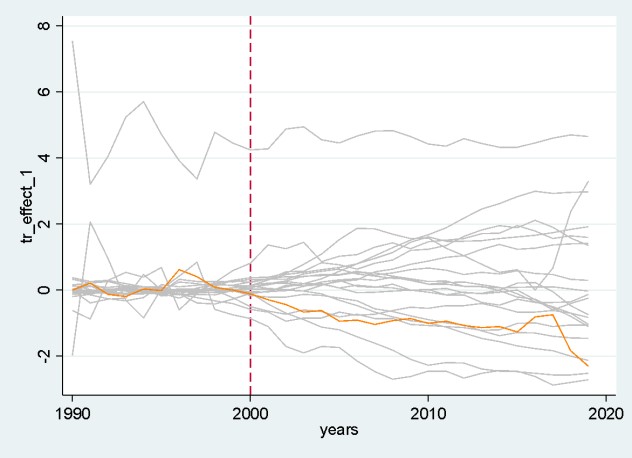


**Figure 1A.** SCGM for CM by congenital diseases in Ghana

**Figure 1B.** Placebo tests for CM by congenital diseases in Ghana

**Figure 1C.** SCGM for CM by asphyxia/trauma in Ghana

**Figure 1D.** Placebo tests for CM by asphyxia/trauma in Ghana

**Notes:** The figure shows the development over time for Ghana and Tanzania and its synthetic control groups on the left. The vertical line shows the implementation of the International Code of Marketing of Breast-Milk Substitutes. On the right we depict the difference between Tanzania and Ghana and its synthetic control groups (orange) and show placebo estimates for the donor pool (gray) to assess statistical significance.

**Figure 2.** SCGM results for overall CM in Ghana and Tanzania.


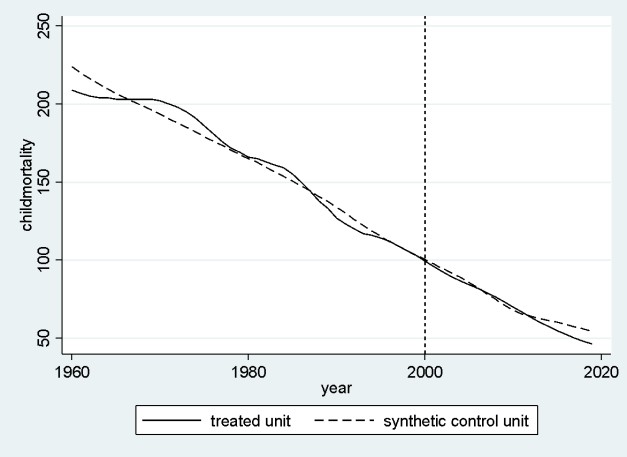

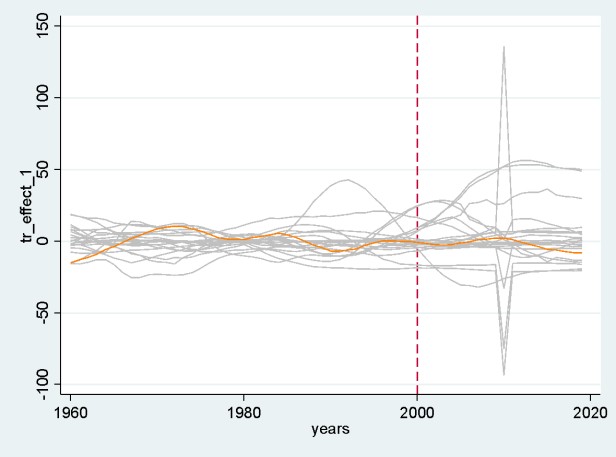


**Figure 2A.** SCGM for CM in Ghana

**Figure 2B.** Placebo tests for CM in Ghana


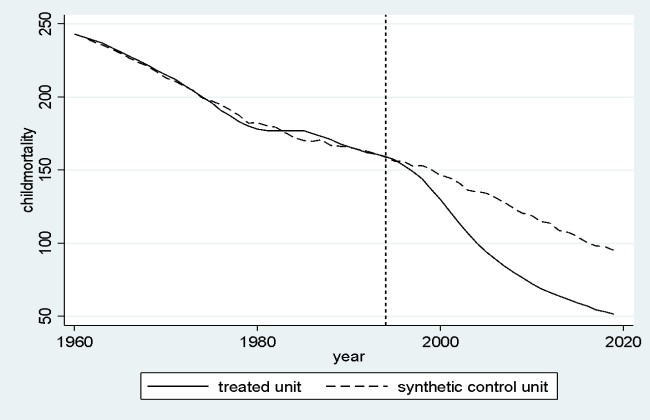

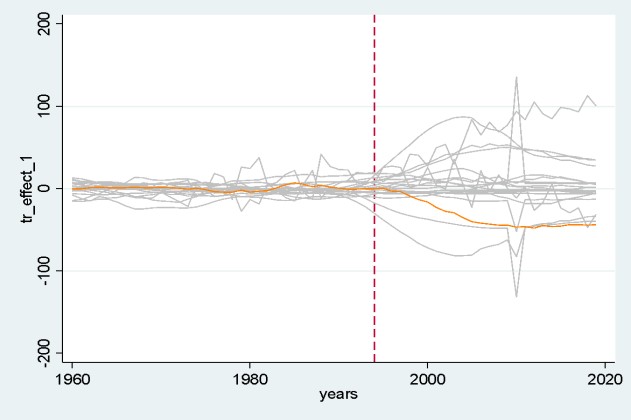


**Figure 2C.** SCGM for CM in Tanzania

**Figure 2D.** Placebo tests for CM in Tanzania

**Notes:** The figure shows the development over time for Ghana and Tanzania and its synthetic control groups on the left. The vertical line shows the implementation of the International Code of Marketing of Breast-Milk Substitutes. On the right we depict the difference between Tanzania and Ghana and its synthetic control groups (orange) and show placebo estimates for the donor pool (gray) to assess statistical significance.
